# Macrophage-derived S100A9 promotes diabetic cardiomyopathy by disturbing mitochondrial quality control via STAT3 activation

**DOI:** 10.7150/ijbs.111128

**Published:** 2025-04-22

**Authors:** Shengqi Huo, Moran Wang, Min Du, Bowen Ren, Tianshu Yang, Lulu Peng, Yue Jiang, Dewei Peng, Lintong Men, Wei Shi, Junyi Guo, Cuntai Zhang, Jiagao Lv, Sheng Li, Li Lin

**Affiliations:** 1 Division of Cardiology, Department of Internal Medicine, Tongji Hospital, Tongji Medical College, Huazhong University of Science and Technology, 1095 Jiefang Avenue, Wuhan, Hubei, China, 430030.; 2 Department of Geriatrics, Tongji Hospital, Tongji Medical College, Huazhong University of Science and Technology, 1095 Jiefang Avenue, Wuhan, Hubei, China, 430030.; 3 Key Laboratory of Vascular Aging, Ministry of Education, Tongji Hospital, Tongji Medical College, Huazhong University of Science and Technology, 1095 Jiefang Avenue, Wuhan, China, 430030.; 4 Department of Cardiology, The Central Hospital of Wuhan, Tongji Medical College, Huazhong University of Science and Technology, Wuhan, China, 430030.

**Keywords:** diabetic cardiomyopathy, macrophage, mitochondrial quality control, S100A9, STAT3

## Abstract

The macrophage-cardiomyocyte crosstalk as a potential intervention target for diabetic cardiomyopathy (DCM) remains deeper exploration. We found S100A9, as an immunoinflammatory mediator, was up-regulated in cardiomyocytes and macrophages in diabetic heart by single-cell analysis. Furthermore, F4/80^+^CCR2^+^S100A9^+^ macrophages in peripheral blood and heart both increased in diabetic mice. S100A9 blocking by paquinimod or macrophage depletion (clodronate) alleviated diabetes-induced cardiac dysfunction, inflammatory macrophage infiltration, serum pro-inflammatory cytokines. More importantly, diabetic cardiac dysfunction, myocardial remodeling, and inflammation could be suppressed by macrophage specific S100A9 knockout (S100a9^flox/flox^Lyz2-Cre). S100A9 activation led to excessive mitochondrial fission, decreased mitophagy flux, and elevated mitochondrial oxidative stress. In addition, proteomics and transcription factor profiling array unveiled S100A9 activated STAT3 in cardiomyocytes. Nevertheless, these effects were mitigated by STAT3(Y705F) mutation, STAT3 knockdown, or paquinimod. Our study highlights macrophage-derived S100A9 as a critical mediator for impaired mitochondrial quality control in diabetic cardiac dysfunction, and targeting S100A9 represents a promising therapeutic target.

## Introduction

Diabetes poses a significant global public health challenge, with projections indicating that an estimated 828 million individuals were affected by the condition in 2022^1^. Diabetic cardiomyopathy (DCM), a specific pathophysiological manifestation of diabetes mellitus, has the potential to culminate in heart failure (HF)^2^. The prevailing mechanisms underlying DCM encompass insulin resistance, excessive oxidative stress, systemic chronic inflammatory activation, neurohumoral homeostasis imbalance, and mitochondrial dysfunction^2, 3^. Research has demonstrated that metabolic imbalances, such as diabetes and obesity, can induce a chronic low-grade inflammation state referred to as para-inflammation^4^. This condition might continuously activate the innate immune system, particularly the monocyte-macrophage system, resulting in detrimental tissue damage. A clinical trial has established a causal link between the mitochondrial activity of peripheral blood mononuclear cells (PBMC) and systemic inflammation in patients with HF^5^. Pertinent research has also confirmed that the PBMC surface marker CD36, which is directly associated with elevated levels of interleukin-6, is significantly increased in patients with type 2 diabetes^6^. This suggests that inflammation response by innate immune system activation is robustly linked to diabetes and heart failure.

S100A9 (S100 calcium binding protein A9) is one of the S100 calcium-binding protein family, alternatively referred to as MRP14 (myeloid-related protein 14)^7^. The primary origins of extracellular S100A9 are myeloid cells, specifically neutrophils, monocytes, and macrophages although other cell types including endothelial cells, and platelets are also capable of secreting S100A9^8^. The S100A9 protein typically exerts its biological function, as a damage-associated molecular pattern (DAMP), through the formation of a heterodimer with S100A8^8^. S100A9 was found to be implicated in various cardiovascular disorders. Clinical research revealed that S100A8/A9 in acute coronary syndrome (ACS) patients were significantly higher than those in patients with normal coronary artery both at local thrombotic sites and systemically^9^. Furthermore, S100A9 plays a crucial role in the conversion of inflammatory monocytes into reparative macrophages in myocardial infarction models by upregulating the levels and activity of the transcription factor Nur77, which could be inhibited by ABR-238901 (or paquinimod, a specific S100A9 blocker)^8^. In lipopolysaccharide(LPS)-induced heart failure, S100A8/A9 was significantly elevated in myocardial tissue, and interacted with the receptor for advanced glycation end products (RAGE), consequently causing a decline in calcium flux and a reduction in myocardial contractility.^10^. Nevertheless, no evidence has been presented to establish a connection between S100A9 and diabetic cardiomyopathy.

## Material and methods

### Mice model

The animal experiments were approved by the institutional review board of Tongji Medical College of Huazhong University of Science and Technology (approval code: 2953), and all procedures complied with the guidelines for the Care and Use of Laboratory Animals of the NIH.

C57BL/6J, db/bks, and db/db mice (male, 6-8 weeks) were offered by the Charles River Laboratory Animal Technology (Beijing, China). The animals were randomly divided into groups: Control, STZ/HFD, STZ/HFD+paquinimod, and db/bks, db/db, db/db+paquinimod. Streptozocin (STZ, S0130, Sigma-Aldrich, Germany) was dissolved by acetate phosphate buffer (C1013, pH 4.5, Solarbio, Beijing, China). STZ/HFD-induced diabetic mice were fed with an 8-week high-fat diet (HFD, D12492, Research Diets, U.S.), and followed by STZ (50 mg/kg per day) intraperitoneal injection for five consecutive days. Mice with blood glucose levels exceeding 15 mmol/L (350 mg/dL) were considered diabetic. Paquinimod (10 mg/kg per day) was administrated daily via intragastric administration for 10 weeks. Mice were sacrificed via intraperitoneal sodium pentobarbital (100 mg/kg) for tissue collection after the cardiac function test.

The cardiac-specific overexpression mice were administered with 1*10^11^ v.g. AAV9-cTNT-S100a9 (Generated by OBiO Technology, Shanghai, China) via tail vein, while the control mice were injected with AAV9-cTNT-GFP. The adeno-associated virus was administered to db/bks and db/db mice twice at 10 and 18 weeks, respectively.

For macrophage depletion, diabetic mice (STZ+HFD) were randomly assigned to intraperitoneally inject 100 μL of 5 mg/mL clodronate liposomes (40337ES08, Yeasen Biotechnology, Shanghai) or control liposomes (PBS) (40338ES08, Yeasen). Over the study period, four such injections were administered to each group.

S100a9-Flox mice (NM-CKO-226344) and Lyz2-Cre mice (NMX-KI-192007) were purchased from Shanghai Model Organisms Center, Inc. Macrophage specific S100A9 knockout mice (S100a9^flox/flox^Lyz2-Cre) was obtained by mating S100a9^flox/flox^ with Lyz2-Cre mice. S100a9^flox/flox^Lyz2-Cre and control mice (S100a9^flox/flox^) were used for diabetic modeling by STZ injection and high-fat diet as previously.

### Bioinformatics analysis

Eight publicly available GEO (Gene Expression Omnibus) datasets of diabetic cardiomyopathy (GSE5606, GSE4745, GSE173384, GSE161052, GSE215979, GSE188418, GSE84796, and GSE26887) were obtained, including mouse, rat, monkey, and human samples. |log_2_(fold change,FC)| > 1 and p-value < 0.05 was used for differentially expressed genes (DEGs) identification via limma package by the R software (version 4.3.2).

Single-cell RNA sequencing dataset (GSE213337) of diabetic cardiomyopathy was obtained from GEO database. This is a publicly available dataset that contains two heart samples. Gene expression data analysis was performed with Seurat R package (version 5.1.0) by the R software. Cells filtration standard: 1 300 < nFeature RNA < 7,000; 2 1000 < nCount RNA < quantile (nCount RNA, 0.97); 3 mitochondrial gene percentage < 20%; 4 erythrocyte gene expression percentage < 5%. The batch effect between samples was eliminated via harmony R package (version 1.2.0). Parameters of dimensionality reduction of 25 and resolution of 0.5 were used for UMAP and tSNE dimensionality reduction analysis. The marker genes in each cluster were identified through Seurat's “FindAllMarkers” function via standards of adjusted p-value<0.05, expression percentage>0.25, and |log_2_FC|>0.25. Pseudotime trajectories analysis was performed via Monocle R package (version 2.30.1). Branched expression analysis modeling function was used for differential expression analysis. The CellChat R package (version 1.6.1) was utilized to infer sophisticated intercellular interactions and construct regulatory networks based on ligand-receptor levels. The p-value<0.05 was used to predict cell-cell interactions between different cell types. The Gene Ontology (GO) in the ClusterProfiler R package (version 4.10.1) was used for enrichment.

### Cell isolation, culture and treatment

Neonatal rat ventricular cardiomyocytes (NRVMs) were isolated by enzymatic digestion following the methods described previously^11^. Isolation of the single cardiomyocyte from adult murine heart of db/db mice by Langendorff perfusion was performed as previously described^12^. Bone marrow-derived macrophages (BMDMs) were prepared as previously described^13^.

AC16, H9c2, and RAW 264.7 cell lines were authenticated using the STR profiling method. Dulbecco's modified Eagle's medium (DMEM, Keygen Biotech, China) supplemented with 10% (v/v) fetal bovine serum (Gibico, Thermo Fisher, U.S.) and 1% (v/v) penicillin/streptomycin (Sangon, China) was used for cell culture. THP-1 cells were authenticated using the STR profiling method. RPMI-1640 medium (Keygen Biotech, China) supplemented with 10% (v/v) fetal bovine serum (Gibico, Thermo Fisher, U.S.) and 1% (v/v) penicillin/streptomycin (Sangon, China) was used for cell culture. THP-1 cells were induced to differentiate into macrophages by culturing them with 100 ng/mL PMA (Phorbol 12-myristate 13-acetate, HY-18739, MCE) for 48 hours. The high glucose concentration was 33 mM, and the incubation time was 24 hours. Mannose (SD8420, Solarbio) was used as the osmotic control.

Recombinant protein S100A9 (rhS100A9) was purified following the expression of an *E. coli* expression vector, achieving a purity exceeding 95% and an endotoxin content below 1 EU/μg (Mabnus Bio, China). Paquinimod (PAQ, HY-100442), lactate (HY-B2227), and Angiotensin II (Ang II, HY-13948) were purchased from MedChemExpress Technology (MCE, China). IL-6 (#200-06), TNFα (#300-01A), and IFNγ (#300-02) recombinant protein were purchased from Pepro Technology (Thermo Fisher, U.S.). Advanced glycation end products (AGE, bs-1158P, Bioss) were purchased from bioss Biochemical Technology (Shanghai, China). Palmitic acid (PA, KC006) was purchased from Kunchuang Biotechnology (Xi'an, China). oxLDL (YB-002) was purchased from Yiyuan Biotechnology (Guangzhou, China).

### Proteomics

Proteomics in AC16 cardiomyocytes exposed to rhS100A9 (2 μg/mL, 24 hours) was performed by BGI Technology (Shenzhen, China). Briefly, proteomics analysis was performed according to steps including protein extraction and digestion, Data-dependent acquisition (DDA) and data-independent acquisition (DIA) analysis by nanoscale liquid chromatography coupled to tandem mass spectrometry, DIA-MS data analysis. Raw data were analyzed using Spectronaut software (12.0.20491.14.21367) with the default settings against the self-built plasma spectral library to achieve deeper proteome quantification. The FDR cutoff for peptide and protein levels was set to 1%. The R package MSstats was used for log2 transformation, normalization, and *p*-value calculation. The criteria was fold change> 2 and *p*< 0.05. The gene set variation analysis (GSVA) package (version 1.50.5) was used for enrichment.

### Transthoracic echocardiography

The cardiac function was measured at baseline and before being sacrificed by VINNO 6 VET (VINNO technology, China). Isoflurane (3% for induction and 1% for maintenance) was used for anesthetization.

### Flow cytometry

Peripheral blood cells were lysed by red blood cell lysate and then resuspended into single-cell suspension. The single cell suspensions were blocked with Purified Rat Anti-Mouse CD16/CD32 (Mouse BD Fc Block, #553141, BD Pharmingen, U.S.), and stained with primary antibodies, including FITC Anti-MouseCD11b (#557396, BD Pharmingen), PerCP-Cy5.5 Anti-Mouse Ly-6C (#560525, BD Pharmingen), Alexa Fluor 647 Anti-Mouse F4/80 (#565853, BD Pharmingen), BV421 Anti-Mouse CD192 (CCR2, #747963, BD Pharmingen), and Alexa Fluor 594 Anti-Mouse S100A9 (NBP2-47980AF594, Novus Biologicals, U.S.). After washing, cells were resuspended in PBS and analyzed on a CytoFLEX-3 flow cytometer (Beckman, U.S.). The data analyses were performed with FlowJo software (TreeStar).

### Transmission Electron Microscopy (TEM)

To distinguish mitochondrial morphologic features of diabetic mice hearts, ultrathin sections of heart tissues were prepared as described previously^14^, and examined by TEM (HT7800/HT7700, HITACHI, Japan).

### HE and fibrotic staining

HE staining kit (G1120, Solarbio, China), Masson-trichrome staining kit (G1340, Solarbio) and Sirius red staining kit (G1472, Solarbio) were used for cardiomyocyte morphology and cardiac fibrosis evaluation.

### Immunofluorescence staining

Cells were fixed with 4% paraformaldehyde for 30 min and treated with 0.5% TritonX-100 for 5 min. After being blocked with 1% goat serum for one hour, anti-pSTAT3 (1:50 dilution, Tyr705, # 9145, CST) was used for staining. Paraffin-embedded heart sections were deparaffinized with xylene and rehydrated in ethanol. 0.1 M citrate buffer (pH 6.0) was used for antigen retrieval. After blocking in 1% goat serum for 1 hour at 37°C, staining was performed as above with anti-S100A9 (1:100 dilution, #73425, CST), anti-α-actinin (1:100 dilution, 66895-1-Ig, Proteintech), anti-CD11b (1:100 dilution, 66519-1-Ig, Proteintech), anti-CD80 (1:100 dilution, M1007-10, HUABIO), anti-F4/80 (1:100 dilution, P47789, Abmart), and anti-CCR2 (1:100 dilution, TD7507M, Abmart) antibodies. Slides were washed and then incubated with FITC Goat Anti-Rabbit IgG (H+L) (AS011, ABclonal) and Cy3 Goat Anti-Mouse IgG (H+L) (AS008, ABclonal). Nuclei were stained with DAPI. The 4-color multiplex immunofluorescence staining kit (MFIHC04A, Abmart) was used in multiplex immunofluorescence staining following the manufacturer's instructions.

### ATP content

ATP content was measured using the ATP assay kit (S0026, Beyotime, China), and normalized by protein concentration.

### Transcription factor profiling array

Transcription factors (TFs) that might be regulated by S100A9 were identified using a TF Activation Profiling Plate Array I (FA-1002, Signosis, U.S.). Briefly, AC16 cardiomyocytes were treated with 2 μg/mL rhS100A9 for 24 h. Nuclear extracts were incubated with biotin-labeled probes which were designed based on the consensus sequences of TF binding sites. The TF-probe complexes were purified, and then the bound probes were separated from the complex, and free oligonucleotides were removed by washing. The detached probes were hybridized in 96-well plates where each well was specifically coated with complementary sequences of the probes. The bound DNA probes were mixed with horseradish peroxidase (HRP)-Streptavidin conjugates, and the luminescence was read in a microplate luminometer (Synergy 2, Bio-Tek Instruments, U.S.).

### Luciferase reporter assay

Dual-Luciferase Reporter Gene Assay Kit (11402ES60; Yeasen) was used for luciferase reporter assay. The wild-type (WT) and mutant promoter of MFF and FIS1 (2.0-kb) were cloned into the pGL3 luciferase reporter vector (Genomeditech, Shanghai, China). Recombinant constructs were co-transfected with pRL-TK plasmid expressing Renilla luciferase (Genomeditech, Shanghai, China) and STAT3(WT)/STAT3(Y705A) overexpressed plasmid (Genomeditech, Shanghai, China) in AC16 cells. After transfection for 48 hours, firefly and Renilla luciferase activities were measured by a microplate luminometer (Synergy 2, Bio-Tek Instruments, U.S.).

### Target genes transfection

Lipofectamine 3000 transfection reagent (L3000008, ThermoFisher, U.S.) and Opti-MEM I reduced serum medium (31985070, ThermoFisher, U.S.) were used for transfection. siRNAs including siNC (siN0000002), siSTIP1 (stB0009775A), siIL6 (stB0006806B), siIL6R (stB0006807A), siIL6ST (stB0006808A), siJAK2 (stB0004834A), and siSTAT3 (stB0000956A) were designed and synthesized by RiboBio(Guangzhou, China). The plasmids of pcDNA3.1(+)-Flag-S100A9, pcDNA3.1(+)-HA-STIP1(Full length, FL), pcDNA3.1(+)-HA-STIP1(Δ259-427), pcDNA3.1(+)-MYC-STAT3(WT), and pcDNA3.1(+)-MYC-STAT3(Y705F) were designed and synthesized by PaiWei Technology (Wuhan, China). The Lentivirus-S100A9-RNAi_1 (#118258-1), Lentivirus-S100A9-RNAi_2 (#118259-31), and Lentivirus-S100A9-RNAi_3 (#118260-14) were constructed by GeneChem Technology (Shanghai, China).

### Western blotting

The whole cell protein extraction and western blotting were performed as previously described^15^. The primary antibodies used were listed as follows: S100A9 (#73425), α-actinin (#6487), STAT3 (#12640), pSTAT3 (Tyr705, #9145), DRP1 (#8570), pDRP1 (Ser616, #4494), and MFF (#84580) were obtained from Cell Signaling Technology (CST, U.S.). S100A8 (15792-1-AP), S100A9 (26992-1-AP), STIP1 (15218-1-AP), FIS1 (10956-1-AP), Tubulin (10068-1-AP), Lamin B (12987-1-AP), GAPDH (10494-1-AP), and HA-tag (51064-2-AP) were obtained from Proteintech Technology (Proteintech, China). S100A9 (A9842), MYC-tag (AE010) was obtained from Abclonal Technology (Abclonal, China).

### Nuclear fractionation extraction

The nuclear extracts were isolated using Nuclear and Cytoplasmic Protein Extraction Kit (KGP150, Keygen Biotech, China) according to the manufacturer's protocol.

### Mitochondrial morphology, superoxide and membrane potential detection

Mito-Tracker Red CMXRos (C1049B, Beyotime, 50nM) was used for mitochondrial morphology analysis. Mitochondrial membrane potential assay kit with JC-1 (C2006, Beyotime, 5μM) was used for mitochondrial membrane potential analysis. MitoBright ROS Deep Red (MT16, DOJINDO, Japan, 5μM) was used for mitochondrial superoxide detection. Images were obtained using a Nikon C2+ confocal microscope (Nikon, Melville, U.S.).

### Mitophagy flux detection

AC16 cells were transfected with adenoviruses encoding mito-Keima (HanBio, Shanghai, China) according to the corresponding instructions. The fluorescence images were obtained using a Nikon C2+ confocal microscope (Nikon, Melville, U.S.), and the 550nm/440nm fluorescence intensity was obtained by a fluorescence microplate reader (Varioskan LUX, thermos scientific).

### Protein-protein docking

Protein structures of STAT3 (1BG1) and STIP1 (7KW7-E) were downloaded from Protein Data Bank (PDB) database. Proteins were pretreated by Discovery Studio software and the ZDOCK module was used to predict protein recognition and interaction based on rigid protein docking algorithm. The docking receptor protein was 1BG1 and the docking ligand protein was 7KW7-E. The Angular Step Size of the docking sampling is set to 6 and the sampling Angle is 15°. 2000 poses are generated as a result of the docking. The pose with a ZDOCK score greater than 17 was selected for further RDOCK analysis to optimize the binding configurations of protein-protein complexes found by ZDOCK. We identified all functional residues according to their interactions using LigPlot+ 2.2.4 software.

### Immunoprecipitation (IP) and IP-MS assays

Whole cell extracts from cells or heart tissues were incubated with primary antibody (MYC, HA, STAT3, or STIP1) or normal immunoglobulin G (IgG) at 4°C overnight. Then, the protein-antibody complex was incubated under agitation with 20 μL protein A/G magnetic beads (RM02915, Abclonal) for 1 h at 4°C. The proteins in the complex were eluted by 20 μL SDS loading buffer and incubated for 10 min at 100°C before separation on SDS-PAGE gel.

To investigate the proteins that interacted with STAT3, we first performed IP in rhS100A9-induced AC16 cardiomyocytes with the anti-STAT3 (#9145, CST). Next, mass spectrometry (MS) was then performed. Briefly, separation was performed by Thermo UltiMate 3000 UHPLC after proteolysis of the IP proteins. The peptides separated by liquid phase chromatography were ionized by a nanoESI source and then passed to a tandem mass spectrometer Q-Exactive HF X (Thermo Fisher Scientific, San Jose, CA) for DDA (Data Dependent Acquisition) mode detection. Statistical analysis of the original mass spectrometric data was performed using Mascot software based on the UniProt database.

### Quantitative real-time polymerase chain reaction (qRT-PCR)

RNA isolation, quality and concentration determination, reverse transcription for cDNA synthesis were performed as described previously^15^. qRT-PCR was performed on StepOnePlus (96-well, Life Technologies, Singapore) real-time PCR system. The mRNA expressions were normalized by housekeeping gene RPS18 or ACTB with the ΔΔCt method. The primer sequences are listed in Table 1.

### ELISA and lipid profiles

Mouse S100A9 ELISA Kit (EK1152, Boster), Mouse S100A8 ELISA Kit (EK1105, Boster), Human S100A9 ELISA Kit (RK09213, Abclonal), and Ultra- Sensitive Mouse Insulin ELISA Kit (#90080, Crystal Chem) were used according to the manufacturer's instruction. Multiple serum inflammatory factors were measured by the ABplex Mouse 20-Plex Panel ELISA Kit (RK04394, Abclonal) or ABplex Mouse 15-Plex Panel ELISA Kit (RK05203, Abclonal). The serum total triglyceride (TG) and total cholesterol (T-CHO) were measured with Chemray 800 auto chemistry analyzer (Rayto Life and Analytical Sciences).

### Statistical analysis

The sample size for each experiment represents the number of independent biological replicates and is provided in the figure legend. The data were presented as the arithmetic means with standard deviation. The unpaired student's t-test with Welch's correction was employed for comparing two groups, while a one-way ANOVA or two-way ANOVA with Bonferroni multiple-comparison correction was used for comparing more than two groups. Statistical significance was determined by *p* < 0.05.

## Results

### S100A9 increases in cardiomyocytes and macrophages of diabetic heart

**Figure 1:**
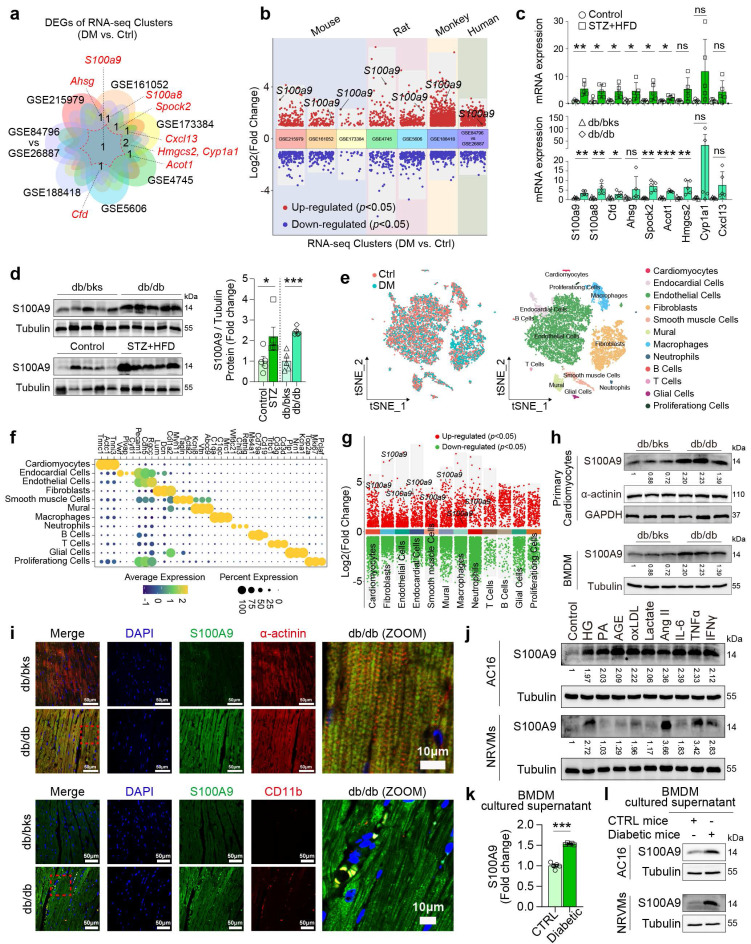
** S100A9 is upregulated in diabetic heart. a-b,** Venn plot and volcano plot of multiple RNA-seq datasets of diabetic heart in GEO database. **c,** mRNA expression of differentially expressed genes (DEGs) in diabetic heart (n=5). **d,** S100A9 protein expression in diabetic heart (n=5). Tubulin was used as a loading control. **e,** tSNE plot of single-cell RNA sequencing dataset (GSE213337). **f,** Dot plot of cell-specific markers. **g,** Volcano plot of DEGs in different cell types. **h,** S100A9 protein expression in primary cardiomyocytes and macrophages (BMDM) of diabetic mice (n=3). **i,** Immunofluorescence staining of diabetic mouse heart tissues for S100A9 (green), α-actinin (red) or CD11b (red). (Scale bar=50 μm, or 10 μm in ZOOM picture). **j,** S100A9 protein expression in cardiomyocytes (AC16 cells, NRVMs) induced by multiple inducing factors. **k,** S100A9 concentration in BMDM cultured supernatant of diabetic mice (n=5). **l,** S100A9 protein expression in AC16 and NRVMs cultured with conditional BMDM supernatant of diabetic mice. *p < 0.05, **p < 0.01, ***p < 0.001. All data are presented as mean ± SD, and statistical significance was determined by unpaired student's t-test. Neonatal rat ventricular cardiomyocytes, NRVMs. Bone marrow-derived macrophages, BMDM. High glucose, HG. Palmitic acid, PA. Advanced glycation endproducts, AGE.

The intersection of eight publicly available GEO datasets of diabetic cardiomyopathy by transcriptomics analysis revealed S100A9 is the unique common up-regulated gene (**Fig. 1a-1b**). In two different types of diabetic mice (STZ+HFD and db/db), S100a9 mRNA expression is both significantly up-regulated (**Fig. 1c**), and protein expression is also increased more than 2-fold in diabetic hearts (**Fig. 1d**). Re-analysis of single-cell transcriptomic data from diabetic hearts (GSE213337) identified twelve distinct cell types based on specific cell markers (**Fig. 1e-1f**). Among these, S100a9 was found increased in multiple cell-types, including cardiomyocytes and myeloid cells (**Fig. 1g**). In primary cardiomyocytes and bone marrow-derived macrophages (BMDM) isolated from diabetic mice, S100A9 expression is increased (**Fig. 1h**). Immunofluorescence staining also reveals that S100A9 co-localizes with myocardial cell marker (α-actinin) or myeloid cell marker (CD11b) in diabetic hearts (**Fig. 1i**).

In AC16 cardiomyocytes and neonatal rat ventricular cardiomyocytes (NRVMs), S100A9 expression was induced by multiple metabolic stressors and inflammatory mediators, including high glucose (HG), advanced glycation endproducts (AGE), oxLDL, Ang II, IL-6, TNFα, and IFNγ (**Figure 1j**). However, the expression profile of S100A9 in cardiomyocytes and macrophages differs to a certain extent. Firstly, the distinct basal expression profiles of S100A9 were evaluated in different cardiomyocytes and macrophages (**Supplementary Figure 1a**). The expression level of S100A9 was relatively low in cardiomyocytes, while macrophages had a high expression of S100A9, indicating that there were differences in the basal expression levels of S100A9 between these two types of cells. Meanwhile, high glucose (33 mM) significantly increased the expression of S100A9 in macrophages (**Supplementary Figure 1b**). More importantly, there are also significant differences in the basal secretion levels of S100A9 between the two types of cells. The basal secretion level of S100A9 in macrophages is higher than that in cardiomyocytes (**Supplementary Figure 1c**). Also, under high glucose stimulation, there was a significant difference in the S100A9 secretion between macrophages and cardiomyocytes. High glucose significantly increased the secretion of S100A9 in macrophages, especially in THP-1 macrophages (**Supplementary Figure 1c**). However, high glucose did not increase the secretion of S100A9 in cardiomyocytes, and there was no significant difference statistically (**Supplementary Figure 1c**). Furthermore, we co-cultured AC16 cardiomyocytes and THP-1 macrophages that were both exposed to high glucose medium. After knockdown of S100A9 in macrophages, the secretion level of S100A9 in the co-cultured supernatant decreased significantly. At the same time, the expression of S100A9 in co-cultured cardiomyocytes also decreased significantly (**Supplementary Figure 1d-1f**). Meanwhile, S100A9 secretion was increased in cultured supernatant of BMDM isolated from diabetic mice (**Figure 1k**). More importantly, S100A9 expression was both induced in AC16 and NRVMs after the conditional supernatant culture of diabetic mice BMDM (**Figure 1l**).

### S100A9 aggravates cardiac dysfunction in diabetic mice and is alleviated by S100A9 blockade

**Figure 2:**
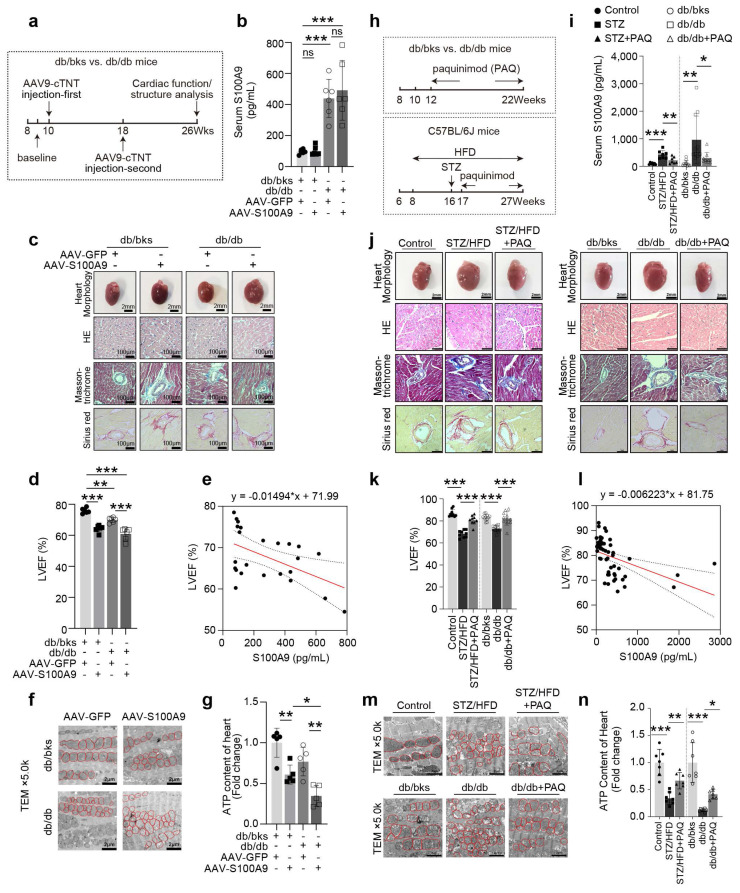
** S100A9 aggravates cardiac dysfunction in diabetic mice and be alleviated by S100A9 blockade. a,** Schematic experimental procedure of AAV9-cTNT-S100A9 overexpression in cardiomyocytes of diabetic mice heart. **b,** Serum S100A9 concentration (n=6) in S100A9-overexpressed diabetic mice. **c,** Heart morphology (Scale bar=2 mm), HE (Scale bar=100 μm), Masson-trichrome (Scale bar=100 μm), and Sirius red (Scale bar=100 μm) staining in S100A9-overexpressed mice. **d,** Cardiac function of LVEF in S100A9-overexpressed diabetic mice (n=6). **e,** Scatter diagram of the correlation between LVEF (%) and serum S100A9 (pg/mL). **f,** Transmission electron microscopy (Scale bar=2 μm) images. **g,** ATP content of diabetic myocardial tissues (n=5). **h,** Schematic experimental procedure of S100A9 blockade by paquinimod (PAQ) in diabetic mice. **i,** Serum S100A9 concentration (n=7, STZ group; n=9, db/db group). **j,** Heart morphology (Scale bar=2 mm), HE (Scale bar=50 μm), Masson-trichrome (Scale bar=50 μm), and Sirius red (Scale bar=50 μm) staining in STZ+HFD and db/db mice. **k,** Cardiac function of LVEF in diabetic mice with PAQ administration (n=9, STZ group; n=11, db/db group). **l,** Scatter diagram of the correlation between LVEF (%) and serum S100A9 (pg/mL) in diabetic mice with PAQ administration. **m,** Transmission electron microscopy (Scale bar=2 μm) images in diabetic mice with PAQ administration. **n,** ATP content of diabetic myocardial tissues (n=7) with PAQ administration. *p < 0.05, **p < 0.01, ***p < 0.001. All data are presented as mean ± SD. Statistical significance was determined by one-way ANOVA.

Cardiac-specific overexpression of S100A9 by tail vein injections of adeno-associated virus (AAV9-cTNT) was performed at the 10th week and 18th week, respectively (**Fig. 2a**). Myocardial overexpression of S100A9 resulted in a considerable increase in heart size in both db/bks and db/db mice (**Fig. 2c, Supplementary Figure 3**), but it had little effect on serum S100A9 levels (**Fig. 2b**). Furthermore, the histological analysis of myocardial tissues using Masson-trichrome and Sirius red staining demonstrates an aggravation of myocardial fibrosis after cardiac-specific overexpression of S100A9 (**Fig. 2c, Supplementary Figure 3**). Meanwhile, a decrease in left ventricular ejection fraction (LVEF), which was inversely correlated with serum S100A9 levels, indicated that cardiac dysfunction was worsened in both diabetic models (**Fig. 2d-2e**). Moreover, the findings from transmission electron microscopy analyses demonstrate an elevation of mitochondrial damage in the myocardial tissues of diabetic mice with S100A9 overexpression (**Fig. 2f**). The ATP content of myocardial tissues both decreases in db/bks and db/db mice with S100A9 overexpression (**Fig. 2g**).

Paquinimod (PAQ) is an S100A9-specific inhibitor by preventing S100A9 binding to TLR4. We administrated paquinimod (10 mg/kg per day) in two different types of diabetic mice (STZ+HFD and db/db) for 10 weeks (**Fig. 2h**). The OGTT (Oral Glucose Tolerance Test), ITT (Insulin Tolerance Test), fasting blood glucose, random blood glucose, insulin levels, total triglyceride (TG), and total cholesterol (T-CHO) for the two models indicated abnormal glucose tolerance, insulin resistance and hyperlipidemia (**Supplementary Figure 2**). The serum S100A9 concentration in diabetic mice is elevated compared with non-diabetic mice, and paquinimod treatment alleviates the increase of S100A9 (**Fig. 2i**). After intervention with the S100A9 inhibitor paquinimod, the S100A8 expression level decreased to a certain extent, but there was no statistical difference in the STZ/HFD-induced diabetic mice (**Supplementary Figure 4a**). Paquinimod also results in a marked reduction in heart size in diabetic mice (**Fig. 2j, Supplementary Figure 3**). HE staining demonstrated reduced myocardial hypertrophy, while Masson-trichrome and Sirius red staining showed alleviation of myocardial fibrosis upon paquinimod treatment (**Fig. 2j, Supplementary Figure 3**). The decrease in systolic function of LVEF in diabetic mice is observed, which could be reversed by paquinimod (**Fig. 2k**). Meanwhile, LVEF level was also negatively and linearly correlated with S100A9 level in paquinimod-treated diabetic mice (**Fig. 2l**). Transmission electron microscopy further revealed increased mitochondrial abundance in diabetic hearts. However, mitochondrial cristae in untreated diabetic mice are bloated, disordered, and fractured, indicating impaired mitochondrial respiration. Notably, these mitochondrial abnormalities are lessened by paquinimod treatment (**Fig. 2m**), and the ATP content in diabetic hearts is also restored after treatment (**Fig. 2n**).

### Macrophage-cardiomyocyte interaction and inflammation increase in diabetic mice

**Figure 3:**
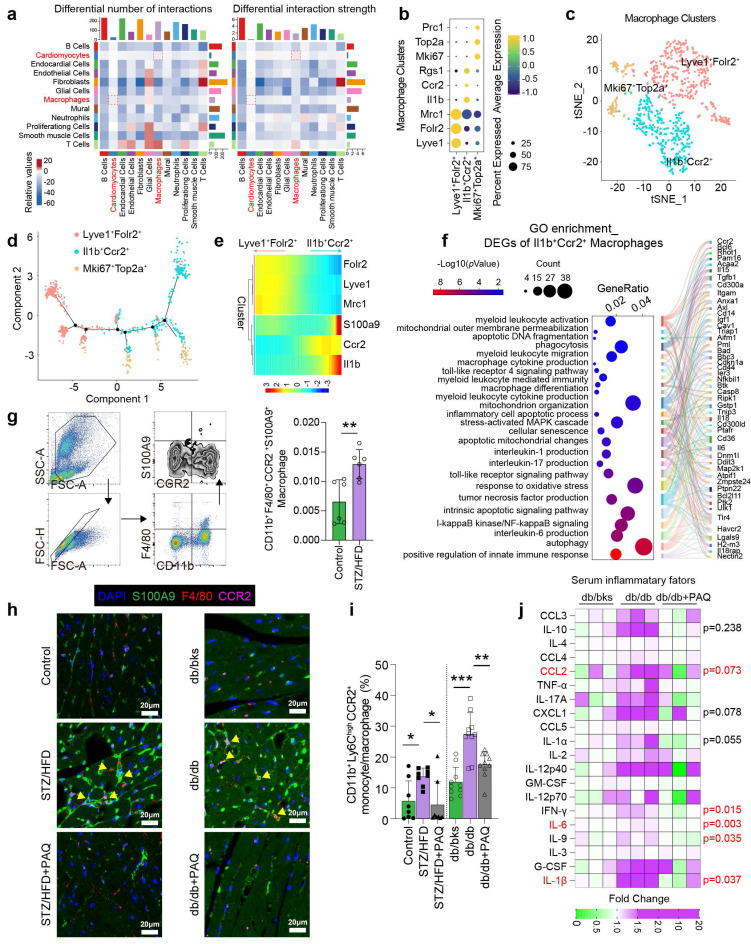
** Macrophage-cardiomyocyte crosstalk and inflammation increase in diabetic mice. a,** Interaction numbers and strength between cell types of scRNA-seq (GSE213337). **b,** Specific markers used for macrophage re-clustering. **c,** Macrophages were re-clustered into three subsets. **d,** The pseudotime distribution of macrophage subtypes by Monocle. **e,** Heatmaps represent the dynamic expression of genes between the Lyve1^+^Folr2^+^ and Il1b^+^Ccr2^+^ clusters. **f,** GO enrichment of DEGs of Il1b^+^Ccr2^+^ cluster. **g,** Flow cytometry plot showing the gating strategy of macrophage in the peripheral blood of diabetic mice, and the percentage of CD11b^+^F4/80^+^CCR2^+^ macrophage in diabetic mice (n=6). **h,** Immunofluorescence staining of diabetic mouse heart for S100A9 (green), F4/80 (red) or CCR2 (violet). (Scale bar=20 μm). **i,** The percentage of CD11b^+^Ly6C^high^CCR2^+^ monocyte/macrophage in diabetic mice (n=8, STZ group; n=10, db/db group). **j,** Serum inflammatory factors levels in db/db mice (n=3 per group). *p < 0.05, **p < 0.01, ***p < 0.001. All data are presented as mean ± SD. Statistical significance was determined by one-way ANOVA.

Single-cell transcriptome analysis reveals that the interaction numbers and strength between multiple cell types increase in diabetic heart, especially between cardiomyocytes and macrophages (**Fig. 3a**). We re-cluster macrophages into three subtypes of Lyve1^+^Folr2^+^, Il1b^+^Ccr2^+^, and Mki67^+^Top2a^+^ by specific cell markers (**Fig. 3b-3c**). The pseudotime distribution of macrophage subtypes shows that Lyve1^+^Folr2^+^ and Il1b^+^Ccr2^+^ differentiate in completely opposing directions (**Fig. 3d**), and dynamic gene expression suggests that S100A9 is mostly expressed in the Il1b^+^Ccr2^+^ cluster (**Fig. 3e**). According to GO enrichment analysis, the differentially expressed genes of Il1b^+^Ccr2^+^ macrophages are significantly enriched in inflammatory and immunological pathways, especially the differentiation, activation, migration, pro-inflammatory cytokine production of myeloid cells (**Fig. 3f**). Flow cytometry confirms a significant increase in the proportion of CD11b^+^F4/80^+^CCR2^+^S100A9^+^ macrophages in the peripheral blood of diabetic mice (**Fig. 3g**). Meanwhile, the number of F4/80^+^CCR2^+^S100A9^+^ macrophages in the diabetic heart is also elevated, and paquinimod obviously reduces their infiltration (**Fig. 3h**). Additionally, paquinimod inhibits the increase of pro-inflammatory subset of CD11b^+^Ly6C^high^CCR2^+^ monocyte/macrophage in diabetic mice (**Fig. 3i**). Serum inflammatory factor levels in db/db mice also indicate that paquinimod substantially reduces inflammation, with the most notable decreases observed in IL-6, IL-1β, IFN-γ, IL-9, IL-1α, CCL2 and CXCL1 (**Fig. 3j**).

### Macrophage depletion by clodronate liposomes ameliorates cardiac dysfunction in diabetic mice

**Figure 4:**
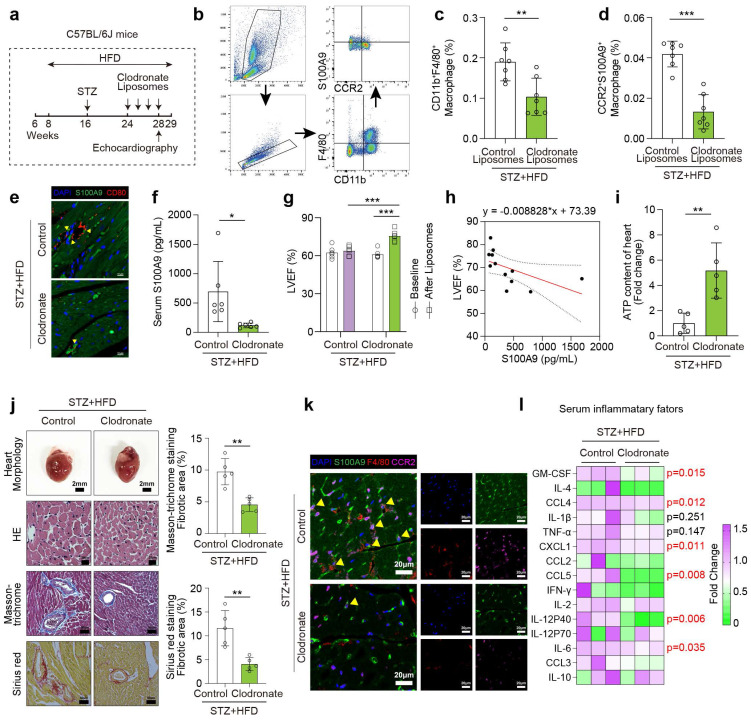
** Macrophage depletion by clodronate liposomes ameliorates cardiac dysfunction in diabetic mice. a,** Schematic experimental procedure of macrophage depletion by clodronate liposomes in diabetic mice. **b,** Flow cytometry plot showing the gating strategy of macrophage in the peripheral blood of diabetic mice. **c-d,** The percentage of CD11b^+^F4/80^+^ macrophage and CD11b^+^F4/80^+^CCR2^+^S100A9^+^ macrophage (n=7). **e,** Immunofluorescence staining for S100A9 (green) and macrophage marker CD80 (red). (Scale bar=10 μm). **f,** Serum S100A9 concentration (n=6). **g,** Cardiac function of LVEF in diabetic mice before and after macrophage depletion (n=6). **h,** Scatter diagram of the correlation between LVEF (%) and serum S100A9 (pg/mL). **i,** ATP content of diabetic myocardial tissues with macrophage depletion (n=5). **j,** Heart morphology (Scale bar=2 mm), HE (Scale bar=20 μm), Masson-trichrome (Scale bar=50 μm), and Sirius red (Scale bar=50 μm) staining. **k,** Immunofluorescence staining of diabetic mouse heart for S100A9 (green), F4/80 (red) or CCR2 (violet). (Scale bar=20 μm).** l,** Serum inflammatory factors levels in diabetic mice with macrophage depletion (n=3 per group). *p < 0.05, **p < 0.01, ***p < 0.001. All data are presented as mean ± SD, and statistical significance was determined by unpaired student's t-test.

To investigate the role of macrophages in diabetic cardiomyopathy, we depleted macrophages in vivo by administering clodronate liposomes once weekly to STZ/HFD-induced diabetic mice for one month (**Fig. 4a**). Flow cytometry and immunofluorescence both indicate macrophages significantly decline after clodronate-liposomes treatment (**Fig. 4b-4c, 4e**). The proportion of CCR2^+^S100A9^+^ macrophages and serum S100A9 level are also reduced by clodronate (**Fig. 4d, 4f**). After clodronate treatment, the decrease of LVEF is mitigated in diabetic mice compared with the baseline (before clodronate, after STZ+HFD induction) and control liposomes group after STZ+HFD induction (**Fig. 4g**). Meanwhile, LVEF level exhibits a negative linear correlation with serum S100A9 level in clodronate-treated diabetic mice (**Fig. 4h**). The decline in ATP content in diabetic hearts is also ameliorated by macrophage depletion (**Fig. 4i**). Enlarged heart size and myocardial fibrosis of diabetic mice are also recovered after clodronate treatment (**Fig. 4j**). Macrophage depletion also alleviates the infiltration of F4/80^+^CCR2^+^S100A9^+^ macrophages in diabetic heart (**Fig. 4k**). Serum inflammatory factor levels in diabetic mice also decrease after clodronate treatment, especially GM-CSF, CCL4, CXCL1, CCL5, IL-12 P40, and IL-6 (**Fig. 4l**).

### Macrophage-specific S100A9 conditional knockout ameliorates cardiac dysfunction in diabetic mice

**Figure 5:**
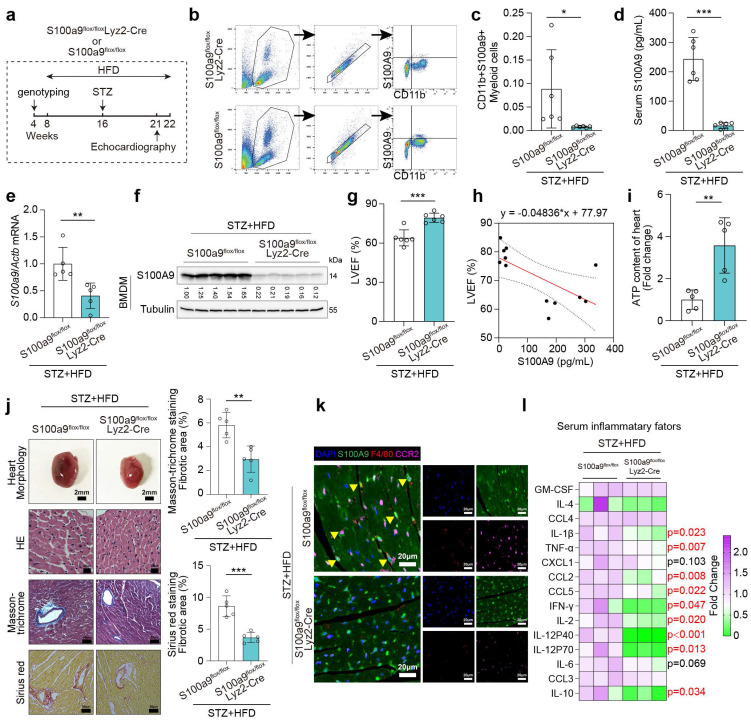
** Macrophage-specific S100A9 conditional knockout ameliorates cardiac dysfunction in diabetic mice. a,** Schematic experimental procedure of macrophage-specific S100A9 conditional knockout (S100a9^flox/flox^Lyz2-Cre) in diabetic mice. **b-c,** Flow cytometry plot showing the gating strategy and the percentage of CD11b^+^S100A9^+^ myeloid cells in the peripheral blood of diabetic mice (n=6). **d,** Serum S100A9 concentration (n=6). **e-f,** S100a9 mRNA and protein expression in BMDM of diabetic mice (n=5).** g,** Cardiac function of LVEF in diabetic mice with macrophage-specific S100A9 knockout (n=6). **h,** Scatter diagram of the correlation between LVEF (%) and serum S100A9 (pg/mL). **i,** ATP content of diabetic myocardial tissues (n=5). **j,** Heart morphology (Scale bar=2 mm), HE (Scale bar=20 μm), Masson-trichrome (Scale bar=50 μm), and Sirius red (Scale bar=50 μm) staining. **k,** Immunofluorescence staining of diabetic mouse heart for S100A9 (green), F4/80 (red) or CCR2 (violet). (Scale bar=20 μm).** l,** Serum inflammatory factors levels in diabetic mice with macrophage-specific S100A9 knockout (n=3 per group). *p < 0.05, **p < 0.01, ***p < 0.001. All data are presented as mean ± SD, and statistical significance was determined by unpaired student's t-test. Bone marrow-derived macrophages, BMDM.

Macrophage-specific S100A9 knockout mice (S100a9^flox/flox^Lyz2-Cre) were obtained by mating S100a9^flox/flox^ with Lyz2-Cre mice. S100a9^flox/flox^Lyz2-Cre and control mice (S100a9^flox/flox^) were utilized to induce diabetes using STZ injection and high-fat diet (**Fig. 5a**). Flow cytometry indicates CD11b^+^S100A9^+^ myeloid cells significantly decline in S100a9^flox/flox^Lyz2-Cre mice (**Fig. 5b-5c**). Moreover, S100A9 mRNA and protein levels in bone marrow-derived macrophages (BMDMs), as well as serum S100A9 levels, are markedly decreased in S100a9^flox/flox^Lyz2-Cre mice (**Fig. 5d-5f**). After knocking out S100A9 in macrophages, the serum S100A8 level decreased to a certain extent, but there was no statistical difference (p = 0.07, **Supplementary Figure 4b**). The protein expression level of S100A8 decreased in the BMDM of macrophage S100a9-knockout mice, while there was no significant change in the myocardial tissue (**Supplementary Figure 4c**). Macrophage-specific S100A9 knockout significantly improves cardiac remodeling and cardiac dysfunction in diabetic mice, as evidenced by enhanced LVEF and reduced cardiac hypertrophy (**Fig. 5g, 5j**). Additionally, LVEF levels show a negative linear correlation with serum S100A9 levels in knockout mice (**Fig. 5h**). ATP content decline in diabetic hearts is also ameliorated by macrophage-specific S100A9 knockout (**Fig. 5i**). Macrophage-specific S100A9 knockout also significantly alleviates the infiltration of F4/80^+^CCR2^+^S100A9^+^ macrophages in diabetic heart (**Fig. 5k**). Multitudinous serum inflammatory factor levels in diabetic mice decline after S100A9 knockout, including IL-1β, TNFα, CCL2, CCL5, IFN-γ, IL-2, IL-12 P40, IL-12 P70, and IL-10 (**Fig. 5l**).

### S100A9 activates STAT3 in cardiomyocytes

**Figure 6:**
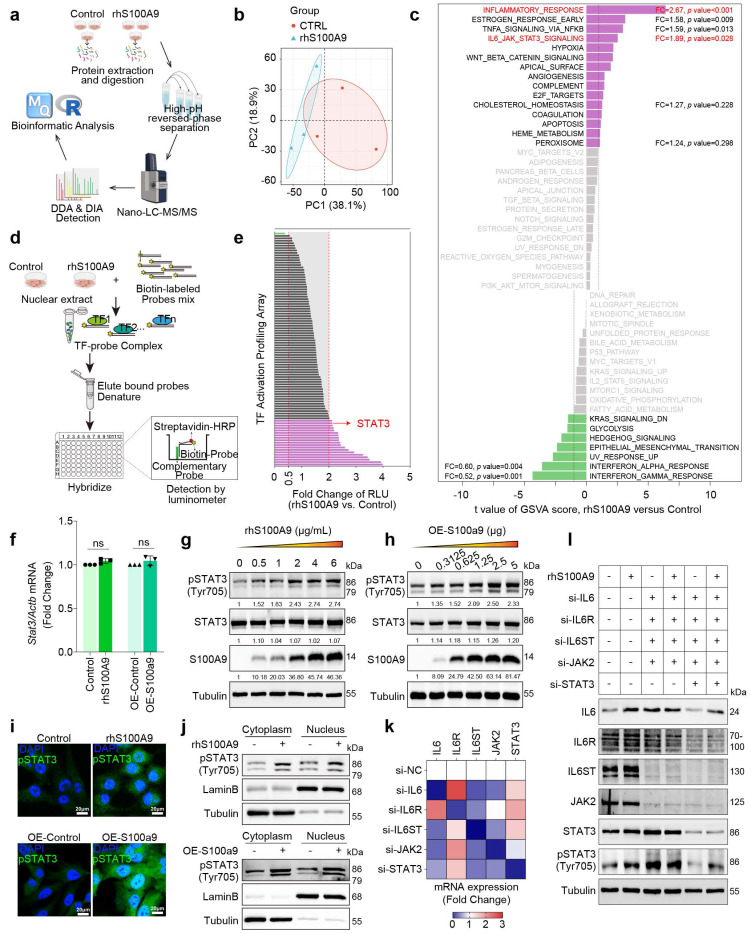
** S100A9 activates STAT3 in cardiomyocytes. a,** DIA-proteomics was performed in AC16 cardiomyocytes exposed to rhS100A9 (2 μg/mL, 24 hours). **b,** PCA plot of proteomics. **c,** GSVA analysis of proteomics. **d,** Workflow for TF promoter-binding profiling plate array in AC16 cardiomyocytes exposed to rhS100A9 (2 μg/mL, 24 hours). **e,** Bar chart of rhS100A9-induced transcription factors (TF) activation in AC16 cardiomyocytes. **f,** STAT3 mRNA expression in AC16 cardiomyocytes induced by rhS100A9 or S100A9 overexpression. **g,** AC16 cells were exposed to concentration gradient of rhS100A9 (0, 0.5, 1, 2, 4, 6 μg/mL) for 24 hours. Representative blots showing the levels of S100A9, STAT3, and phosphorylation of STAT3 (Tyr705). Tubulin was used as a loading control. **h,** AC16 was transfected with a concentration gradient of pcDNA3.1(+)-Flag-S100A9 plasmid (0, 0.3125, 0.625, 1.25, 2.5, 5 μg DNA) or empty vector plasmid for 48 hours. Representative blots showing the levels of S100A9, STAT3, and pSTAT3 (Tyr705). Tubulin was used as a loading control. **i,** Immunofluorescence of pSTAT3 (Tyr705) in AC16 cells (Scale bar = 20 μm). **j,** Nuclear fraction of AC16 cells with rhS100A9 exposure or S100A9 overexpression was separated. Western blots detected pSTAT3 (Tyr705). Lamin B and Tubulin were used as nuclear and cytoplasmic markers, respectively. **k,** IL6, IL6R, IL6ST, JAK2, and STAT3 mRNA level in AC16 cells after siRNA transfection. **l,** Representative blots showing the levels of IL6, IL6R, IL6ST, JAK2, STAT3 and pSTAT3 (Tyr705) in AC16 cardiomyocytes with siRNA transfection and/or rhS100A9 incubation. Tubulin was used as a loading control. All data are presented as mean ± SD. Statistical significance was determined by unpaired student's t-test.

To investigate the specific biological impact of S100A9, proteomics analysis was performed on AC16 cardiomyocytes exposed to rhS100A9 (2 μg/mL, 24 hours) (**Fig. 6a**). A total of 6,786 proteins are identified using LC-MS, and principal component analysis (PCA) reveals significant differences between the treated and control groups (**Fig. 6b**). GSVA enrichment analysis hints that differentially expressed proteins are markedly enriched in the inflammatory response and the IL6/JAK/STAT3 signaling pathway (**Fig. 6c**). To identify the underlying transcriptional mediators that affect rhS100A9-induced gene transcription, TF promoter-binding profiling plate array analysis is performed in AC16 cardiomyocytes exposed to rhS100A9 (2 μg/mL, 24 hours) (**Fig. 6d**). The relative luminescence unit (RLU) of STAT3 after rhS100A9 exposure is upregulated more than 2-fold compared with control group in AC16 cardiomyocytes (**Fig. 6e**). In addition, rhS100A9 exposure and S100A9 overexpression both increase phosphorylation of STAT3(Tyr705) in AC16 cells without altering STAT3 mRNA expression (**Fig. 6f-6h**). The nuclear localization and activation of pSTAT3(Tyr705) are further confirmed, as evidenced by increased nuclear levels of pSTAT3(Tyr705) and elevated nuclear fluorescence intensity following rhS100A9 or S100A9 overexpression in AC16 cells (**Fig. 6i-6j**). After knocking down the crucial genes in IL-6/JAK/STAT3 pathway, the induction of pSTAT3(Tyr705) by rhS100A9 is not completely inhibited (**Fig. 6k-6l**), indicating there are other mediators involved in the S100A9-induced STAT3 activation.

### S100A9 promotes STIP1-STAT3 interaction in cardiomyocytes and diabetic heart tissues

**Figure 7:**
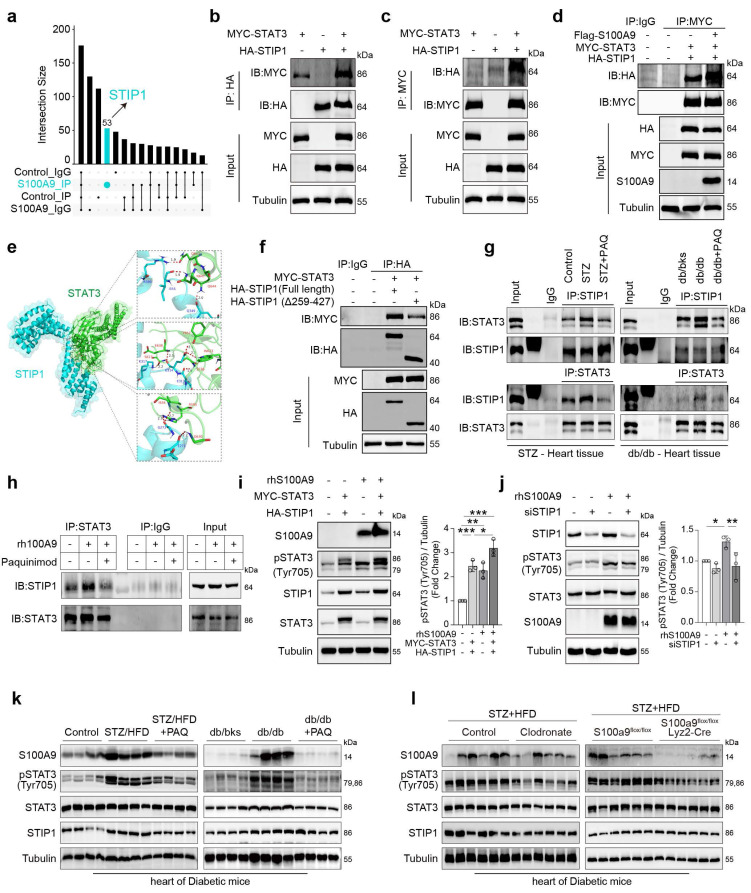
** S100A9 promotes STIP1-STAT3 interaction in cardiomyocytes and diabetic heart tissues. a,** Upset-Venn plot of IP-MS results in rhS100A9-treated AC16 cardiomyocytes. **b-c,** co-immunoprecipitation (co-IP) of MYC-STAT3 and HA-STIP1 in AC16 cardiomyocytes. **d,** S100A9 promoted STIP1 binding with STAT3 in AC16 cells transfected with Flag-S100A9, MYC-STAT3 and HA-STIP1. **e,** The predicted binding sites between STIP1 and STAT3. Optimized pose model of STIP1-STAT3 interaction by R-Dock analysis.** f,** Full-length STIP1 or truncated mutant STIP1 (TPR5-8 domain, Δ259-427) were co-transfected with MYC-STAT3 in AC16 cardiomyocytes. co-IP of STAT3 and STIP1 was perform with HA-tag antibody. **g,** co-IP of STAT3 and STIP1 was performed in STZ-induced or db/db diabetic heart tissues treated with/without paquinimod (PAQ). **h,** co-IP of STAT3 and STIP1 in AC16 cardiomyocytes incubated with rhS100A9 and paquinimod. **i,** Representative blots showing the levels of pSTAT3(Tyr705) in AC16 cells with STAT3 and STIP1 overexpression and/or rhS100A9 exposure (n=3). **j,** Representative blots showing the levels of pSTAT3(Tyr705) in AC16 cells with STIP1 knockdown and/or rhS100A9 exposure (n=3). **k,** Representative blots showing the protein expression of S100A9, pSTAT3 (Tyr705), STAT3, and STIP1 in STZ-induced or db/db diabetic heart tissues treated with/without paquinimod (PAQ) (n=4). Tubulin was used as a loading control. **l,** Representative blots showing the S100A9, pSTAT3 (Tyr705), STAT3, and STIP1 protein expression in heart tissues of diabetic mice with macrophage depletion or macrophage-specific S100A9 conditional knockout (n=6 per group). Tubulin was used as a loading control. *p < 0.05, **p < 0.01, ***p < 0.001. All data are presented as mean ± SD. Statistical significance was determined by one-way ANOVA.

Immunoprecipitation (IP) was performed using an anti-STAT3 antibody in rhS100A9-induced AC16 cells, and the immunoprecipitates were analyzed by mass spectrometry (MS). The Upset Venn plot shows that STIP1 is one of the identified unique proteins that could interact with STAT3 after rhS100A9 exposure (**Fig. 7a**). The interaction between STIP1 and STAT3 is confirmed through immunoprecipitation in AC16 cells that overexpress MYC-STAT3 and/or HA-STIP1 (**Fig. 7b-7c**). Notably, S100A9 overexpression increased the interaction between STAT3 and STIP1 (**Fig. 7d**). The protein-to-protein docking using Z-DOCK module in Discovery Studio software, further refined by the R-DOCK method, predicts that the critical amino acid residues of STIP1 protein for its binding with STAT3 are mainly located in TPR 5-8 domain (**Fig. 7e**). The absence of the TPR 5-8 domain in mutant STIP1 results in a reduced STIP1-STAT3 interaction compared with the overexpression of full-length STIP1 (**Fig. 7f**). In STZ/HFD-induced or db/db diabetic heart tissues, the interaction of STIP1 and STAT3 is enhanced, whereas it is weakened by paquinimod administration (**Fig. 7g**). Paquinimod also decreases the STIP1-STAT3 interaction in rhS100A9-induced AC16 cardiomyocytes (**Fig. 7h**). STIP1 overexpression in AC16 cells induces phosphorylation of STAT3(Tyr705), which was further enhanced by rhS100A9 treatment (**Fig. 7i**). In contrast, STIP1 knockdown reduces the elevated pSTAT3(Tyr705) in rhS100A9-induced AC16 cells (**Fig. 7j**). Immunoblot analyses demonstrate a notable increase in the expression of S100A9 and pSTAT3(Tyr705) in diabetic hearts, which were successfully reversed by paquinimod (**Fig. 7k**). Similarly, the elevation of S100A9 and pSTAT3 (Tyr705) in diabetic hearts could be alleviated by macrophage depletion or macrophage-specific S100A9 conditional knockout (**Fig. 7l**).

### S100A9 promotes excessive mitochondrial fission and inhibits mitophagy flux in cardiomyocytes

**Figure 8:**
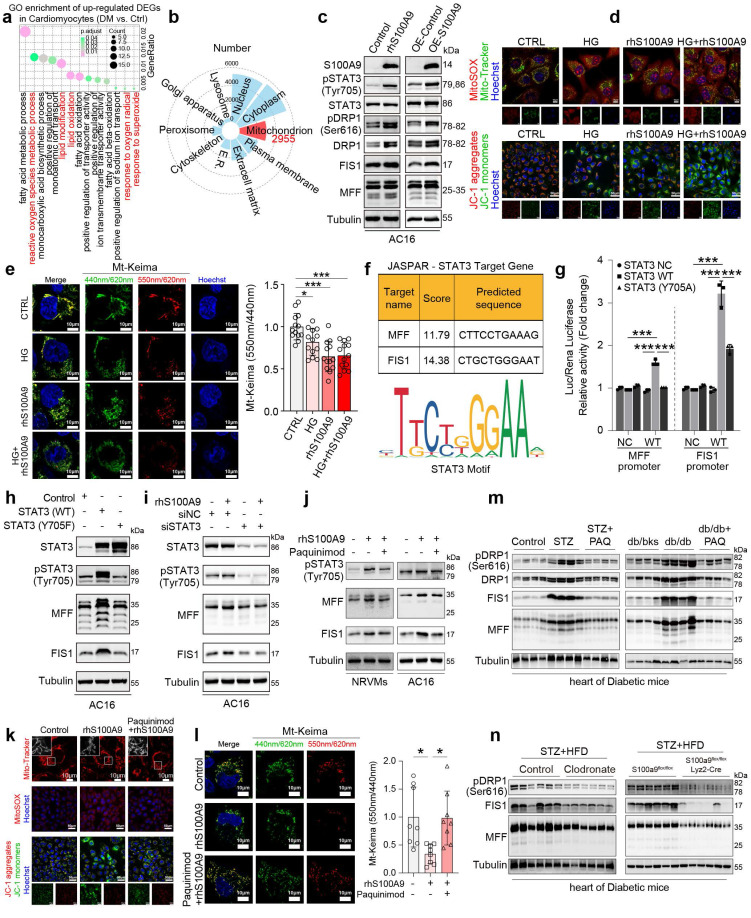
** S100A9 promotes excessive mitochondrial fission and inhibits mitophagy flux in cardiomyocytes. a,** GO enrichment of up-regulated DEGs in cardiomyocytes of scRNA-seq (GSE213337). **b,** Subcellular localization of the identified differentially expressed proteins by proteomics in AC16 cells exposed to rhS100A9. **c,** Protein expression of S100A9, pSTAT3 (Tyr705), STAT3, pDRP1 (Ser616), DRP1, FIS1, and MFF in AC16 cells with S100A9 overexpression or rhS100A9 exposure. **d,** Mito-Tracker, MitoSOX and JC-1 staining in AC16 cardiomyocytes exposed to high glucose (HG) and/or rhS100A9 (Scale bar=10 μm or 50 μm). **e,** Mt-Keima staining and the ratio of 550nm/440nm fluorescence intensity (n=13) detected by microplate reader in AC16 cardiomyocytes exposed to high glucose and/or rhS100A9 (Scale bar=10 μm). **f,** Target genes of STAT3 predicted by JASPAR database. **g,** Dual-luciferase reporter assay. **h,** Representative blots showing the levels of STAT3, pSTAT3 (Tyr705), MFF, and FIS1 in AC16 cells overexpressed with wild-type STAT3 (WT) or mutant STAT3 (Y705F). **i,** Representative blots showing the levels of pSTAT3 (Tyr705), STAT3, MFF, and FIS1 in AC16 cells with STAT3 knockdown and/or rhS100A9 exposure. **j,** Representative blots showing the levels of pSTAT3 (Tyr705), MFF, and FIS1 in NRVMs and AC16 cardiomyocytes with rhS100A9 and/or paquinimod exposure (20μM, 24 hours). **k,** Mito-Tracker, MitoSOX and JC-1 staining in AC16 cardiomyocytes treated with rhS100A9 and/or paquinimod (Scale bar = 10 μm or 50 μm). **l,** Mt-Keima staining and the ratio of 550nm/440nm fluorescence intensity (n=8) detected by microplate reader in AC16 cardiomyocytes exposed to rhS100A9 and/or paquinimod. **m,** Representative blots showing the protein expression of pDRP1 (Ser616), DRP1, FIS1 and MFF in STZ-induced or db/db diabetic heart tissues treated with/without paquinimod (PAQ) (n=4). **n,** Representative blots showing the pDRP1 (Ser616), FIS1 and MFF protein expression in heart tissues of diabetic mice with macrophage depletion or macrophage-specific S100A9 conditional knockout (n=6 per group). *p < 0.05, **p < 0.01, ***p < 0.001. All data are presented as mean ± SD. Statistical significance was determined by one-way ANOVA (**e, l**), or two-way ANOVA (**g**).

Single-cell RNA sequencing (scRNA-seq) analysis of diabetic cardiomyocytes revealed that the upregulated differentially expressed genes (DEGs) were enriched in oxidative stress-related pathways (**Fig. 8a**). Meanwhile, the subcellular localization analysis of the identified differentially expressed proteins in rhS100A9-induced AC16 cardiomyocytes revealed that 2955 proteins are possibly mitochondria-localized proteins (**Fig. 8b**). Notably, exposure to rhS100A9 or S100A9 overexpression upregulated the expression of pSTAT3(Tyr705), pDRP1(Ser616), DRP1, FIS1, and MFF in AC16 cardiomyocytes (**Fig. 8c**). The mitochondrial fission factor (MFF) and Mitochondrial fission 1 protein (FIS1) are critical receptors that recruit DRP1 to fission sites. Hence, the upregulation of pDRP1(Ser616), DRP1, FIS1 and MFF indicates that S100A9 results in excessive mitochondrial fission. The Mito-tracker staining validates the mitochondrial fragmentation, which demonstrated that rhS100A9 and/or high glucose results in a rounded fragmentation of the mitochondria and less connected mitochondrial network in AC16 cardiomyocytes, accompanied with increased mitochondrial oxidative stress (**Fig. 8d, Supplementary Figure 5a**). Meanwhile, the JC-1 aggregates (red fluorescence) decreased and monomers increased (green fluorescence) after rhS100A9 and/or high glucose incubation. This indicates rhS100A9 and/or high glucose decreased mitochondrial membrane potential in AC16 cardiomyocytes (**Fig. 8d, Supplementary Figure 5b**). To further detect mitophagy flux, Mt-Keima adenoviruses transfection is conducted. When autophagosomes fuse with acidified lysosomes to form autolysosomes, Mt-Keima manifests red fluorescence. Mt-Keima detection reveals that rhS100A9 treatment reduces the number of Keima dots (red, autolysosomes) and decreases the 550 nm/440 nm fluorescence intensity ratio in AC16 cells (**Fig. 8e**). This indicates rhS100A9 supressed mitophagy flux of AC16 cardiomyocytes. In addition, high glucose exposure further aggravated the intracellular accumulation of functionally impaired mitochondria and the decrease of mitophagy flux (**Fig. 8d-8e**).

The dual-luciferase reporter assay demonstrates a statistical increase in MFF and FIS1 promoter activity in AC16 cells upon overexpression of wild-type STAT3 (STAT3 WT) rather than the dominant-negative mutant STAT3 (STAT3-Y705A) (P<0.05) (**Fig. 8f-8g**). Moreover, in AC16 cells, the overexpression of wild-type STAT3 increases the protein expression of pSTAT3(Tyr 705), as well as MFF and FIS1. Conversely, mutant STAT3 (Y705F) hinders the phosphorylation of STAT3(Tyr 705) and suppresses the protein expression of MFF and FIS1 (**Fig. 8h**). Meanwhile, the knockdown of STAT3 almost reverses the upregulation of pSTAT3(Tyr705), MFF, and FIS1 induced by rhS100A9 (**Fig. 8i**). In the NRVMs and AC16 cardiomyocytes, paquinimod inhibits the expression of pSTAT3(Tyr705), MFF and FIS1 induced by rhS100A9 (**Fig. 8j**). Additionally, paquinimod alleviates abnormal mitochondrial fragmentation, increased mitochondrial oxidative stress, and reduced mitochondrial membrane potential induced by rhS100A9 (**Fig. 8k-8l, Supplementary Figure 5c-5d**). More importantly, the decreased 550 nm/440 nm fluorescence intensity ratio was reversed by paquinimod, indicating impaired mitophagy flux induced by rhS100A9 in AC16 cardiomyocytes was suppressed by paquinimod (**Fig. 8l**). Immunoblot analysis further confirmed that the expression of mitochondrial fission-related factors, such as pDRP1(Ser616), DRP1, MFF, and FIS1 were elevated in diabetic hearts. Remarkably, macrophage depletion or macrophage-specific S100A9 conditional knockout effectively reverses these changes (**Fig. 8m-8n**).

## Discussion

In the present study, we identified S100A9 as a critical mediator in the pathogenesis of diabetic cardiomyopathy (DCM). Specifically, we demonstrated that S100A9 is upregulated in myocardial tissue from diabetic models, with elevated expression in both macrophages and cardiomyocytes, but the basal expression and secretion levels of S100A9 between the two types of cells are different. Co-cultured cardiomyocytes and macrophages suggested that macrophages are an important source of S100A9 secretion, and S100A9 derived from macrophages has a significant inducing effect on cardiomyocytes. Importantly, single-cell transcriptomic analysis further revealed a significant intercellular interaction between these two cell types. S100A9-targeted interventions, including S100A9 inhibitor paquinimod, macrophage depletion via clodronate liposomes and macrophage-specific S100A9 conditional knockout, effectively alleviated myocardial injury and improved cardiac function in diabetic mice. Mechanistically, S100A9 activated the STAT3 signaling pathway, leading to mitochondrial dysfunction characterized by increased fission, decreased mitophagy, and impaired mitochondrial quality control. These mitochondrial alterations resulted in disrupted cardiomyocyte energy metabolism, thereby exacerbating cardiac dysfunction. Collectively, our findings underscore the pivotal role of S100A9 in the progression of DCM and highlight its therapeutic potential. S100A9 has been studied in various cardiovascular diseases, such as atherosclerosis^16^ and myocardial infarction. However, its role in diabetic cardiac injury has not been previously reported. To our knowledge, only one previous study in diabetic hearts illustrated the relationship of S100A8/A9 and doxorubicin-induced cardiotoxicity, and without more mechanism exploration and target intervention^17^. This study is the first to directly implicate S100A9 in DCM, expanding its known roles in cardiovascular pathophysiology.

S100A9 is mainly secreted by myeloid cells, and plays a crucial role in regulating inflammatory response and maintaining immune homeostasis. There are several studies reported S100A9 blockage promoting the improvement in diabetic associated diseases, such as diabetic nephropathy, microcalcification and chronic wounds in diabetes mice. In diabetic nephropathy mice, S100A8/A9 inhibited by lentivirus transfected into the mice kidneys ameliorated renal interstitial fibrosis^18^. S100A9 inhibitor, paquinimod attenuated retinal injuries in diabetic mice^19^. Macrophage-targeted nanoparticles coated with S100A9 siRNA decreased inflammation and microcalcification in diabetic mice^20^. S100A9 blockage restored fibroblast ECM functions and promoted tissue repair in db/db mice^21, 22^.

However, it is unclear how inflammation dysregulation and immune disturbance lead to cardiomyocyte injury. Based on the findings, we further investigated the derivation of S100A9 and focused on the crosstalk between cardiomyocytes and macrophages. Importantly, we found the subset of F4/80^+^CCR2^+^S100A9^+^ macrophages enriched in diabetic heart and peripheral blood. To thoroughly elucidate the role of S100A9 derived from macrophages and decrease the confounding factors induced by single intervention, we gradually evaluated the effects of S100A9 inhibitor, macrophage depletion and macrophage-specific S100A9 knockout in diabetic cardiomyopathy mice. These interventions demonstrated therapy effects on the development of diabetic cardiomyopathy. These data suggest that macrophage-derived S100A9 contributes to DCM progression primarily through inflammatory pathways.

As a charming transcriptional factor, STAT3 widely takes part in many cellular processes, including inflammatory responses, energy metabolism and cellular viability. Our results indicate that there are other pathways to activate STAT3 independent of the IL6/IL6R/IL6ST/JAK2 signaling pathway. According to the reports, the non-canonical pathway, likely involving TLR4 and Src kinases, takes over STAT3 activation when IL-6/JAK2 signaling is blocked. Specifically, rhS100A9 activates TLR4, which signals through MyD88 and Src to phosphorylate STAT3, bypassing JAK2^23-27^. We want to explore other signaling pathways for STAT3 activation, and STIP1 was discovered through mass spectrometry detection. The interplay between S100A9, IL-6 signaling, and STIP1-STAT3 binding is complex. Under normal conditions, IL-6 stimulates S100A9 transcription via STAT3, and S100A9 further induces IL-6 expression, forming a feedback loop^28, 29^. In this study, we observed increased STAT3 activation following rhS100A9 treatment, in the context of IL-6 signaling inhibition, and propose a novel mechanism of S100A9 promoting STIP1-STAT3 binding, which further leads to STAT3 activation. Hence, the fact that rhS100A9 stimulates IL-6 signaling or promotes STIP1-STAT3 binding, both of which lead to p-STAT3 (Tyr705) activation, represents neither a juxtaposition nor a temporal relationship. The crosstalk between signaling pathways is also a complex and interesting topic, which is worthy of evaluation and elucidating STIP1-STAT3 binding and IL-6 signaling pathway crosstalk in cardiomyocytes in the future.

The role of STAT3 in regulating mitochondrial fission and fusion, as well as its impact on mitophagy, has been previously reported^30-32^. STAT3 activation has been found to induce MFN2 in doxorubicin-induced cardiotoxicity and OPA1 in the transverse aortic constriction model^33, 34^. Meanwhile, our group has reported that STAT3 regulates mitophagy proteins and mitochondrial function^30^ in colon cancer. In hepatocellular carcinoma and sepsis-induced cardiomyopathy, S100A9 exacerbates the disease by promoting mitochondrial fission through the deubiquitination of PGAM5 or the activation of ERK1/2-DRP1, respectively^35, 36^. However, our study elucidates a novel S100A9-STAT3 signaling axis that regulates mitochondrial quality control in cardiomyocytes. Activation of STAT3 by S100A9 disrupted mitochondrial dynamics by promoting excessive fission, reducing mitophagy, and impairing mitochondrial homeostasis. Our study underscores the new insight of the STIP1/STAT3/MFF-FIS1 signaling axis in regulating mitochondrial quality control in the context of DCM. Furthermore, these investigations collectively highlight that targeting the S100A9-STAT3 axis may therefore represent a novel therapeutic avenue for restoring mitochondrial function and metabolic homeostasis in diabetic hearts.

Certainly, there are also some limitations at present. It is also important to explore whether the S100A9 derived from cardiomyocytes themselves plays a role in diabetic cardiomyopathy. Whether S100A9 from other cell sources, such as cardiac fibroblasts or adipocytes in epicardial adipose tissue, also plays a crucial role in diabetic cardiomyopathy remains unknown. It is still uncertain whether S100A9 from more tissue sources exerts similar regulatory effects. Meanwhile, through what molecular mechanisms S100A9 regulates the inflammatory response process of macrophages is also an unknown issue that requires in-depth exploration in the future. The molecular bases of S100A9-mediated mitochondrial dysfunction and its wider significance in diabetes complications should be further investigated in future studies.

## Conclusions

In conclusion, our study identifies S100A9 as a critical mediator of DCM through its regulation of macrophage-cardiomyocyte crosstalk by exacerbating inflammation and disturbing mitochondrial quality control. Therapeutic strategies targeting S100A9, including pharmacological inhibition and genetic modification significantly improved cardiac outcomes, highlighting its clinical translational potential.

## Supplementary Material

Supplementary figures.

## Figures and Tables

**Table 1:** The primer sequences.

Gene	Forward	Reverse
Rps18 (mouse)	AGGATGTGAAGGATGGGAAG	TTCTTCAGCCTCTCCAGGTC
Actb (mouse)	GTGCTATGTTGCTCTAGACTTCG	ATGCCACAGGATTCCATACC
S100a9 (mouse)	GGCCAACAAAGCACCTTCTC	GGCTTCATTTCTCTTCTCTTTCTTC
S100a8 (mouse)	ACTTCGAGGAGTTCCTTGCG	TACTCCTTGTGGCTGTCTTTGT
Cfd (mouse)	AGCCGACCTGACAGCCTTGAG	CAACCAGCCACGTCGCAGAG
Ahsg (mouse)	TGCAGAGGACGTTCGTAAGTTGTG	GGCAGTGTTGACGGTGTGGAC
Spock2 (mouse)	TGCTGAGTGGTGCTTCTGTTTCTG	TGGCTGCCTCCTGGATCTGTG
Acot1 (mouse)	GCTGGCTGGGAAGGGCTTTG	CGCAGGTAGTTCACGGCTTCTTC
Hmgcs2 (mouse)	GCAGCCTACCGCAAGAAGATCC	AACATCAACCGAGCCAGGGATTTC
Cyp1a1 (mouse)	TGGAGCCTCATGTACCTGGTAACC	CTGCCGATCTCTGCCAATCACTG
Cxcl13 (mouse)	TGTGTGAATCCTCGTGCCAAATGG	GAGCTTGGGGAGTTGAAGACAGAC
ACTB (human)	CCTGGCACCCAGCACAAT	GGGCCGGACTCGTCATAC
IL6 (human)	TGAACTCCTTCTCCACAAGCG	GATGCCGTCGAGGATGTACC
IL6R (human)	TCCAGCATCACTGTGTCATCC	CTGGATTCTGTCCAAGGCGT
IL6ST (human)	AACAGCATCCAGTGTCACCTT	TCCCCTCGTTCACAATGCAA
JAK2 (human)	AGCAAGCAAACCAAGAGGGT	GGGCCATGACAGTTGCTTTG
STAT3 (human)	AGCAGCACCTTCAGGATGTC	GCATCTTCTGCCTGGTCACT
