# Supplementary material for: Macrophage-derived S100A9 promotes diabetic cardiomyopathy by disturbing mitochondrial quality control via STAT3 activation
Source: Int J Biol Sci. 2025 Apr 22;21(7):3061–80. doi: 10.7150/ijbs.111128 (PMC12080395; doi:10.7150/ijbs.111128)
Supplement: Supplementary file 1 — Supplementary figures. [file ijbsv21p3061s1.pdf]

Supplementary Figure 1

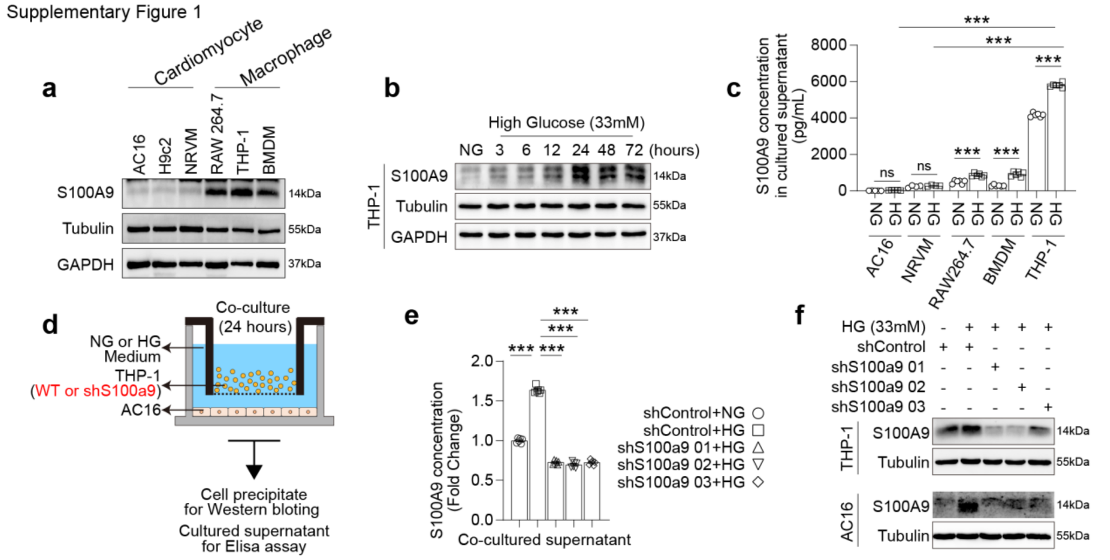

Supplementary Fig. 1 Differences in the expression and secretion of S100A9 in macrophages and cardiomyocytes.

a, S100A9 expression in macrophages and cardiomyocytes. **b,** S100A9 expression in THP-1 macrophages exposed to high glucose (33mM). **c,** S100A9 secretion in macrophages and cardiomyocytes exposed to high glucose (33mM). **d,** schematic diagram of co-cultured macrophages and cardiomyocytes. **e,** S100A9 level in co-cultured supernatant. **f,** S100A9 expression in co-cultured macrophages and cardiomyocytes. **p* < 0.05, ***p* < 0.01, ****p* < 0.001. All data are presented as mean ± SD. Statistical significance was determined by one-way ANOVA.

1

Supplementary Figure 2

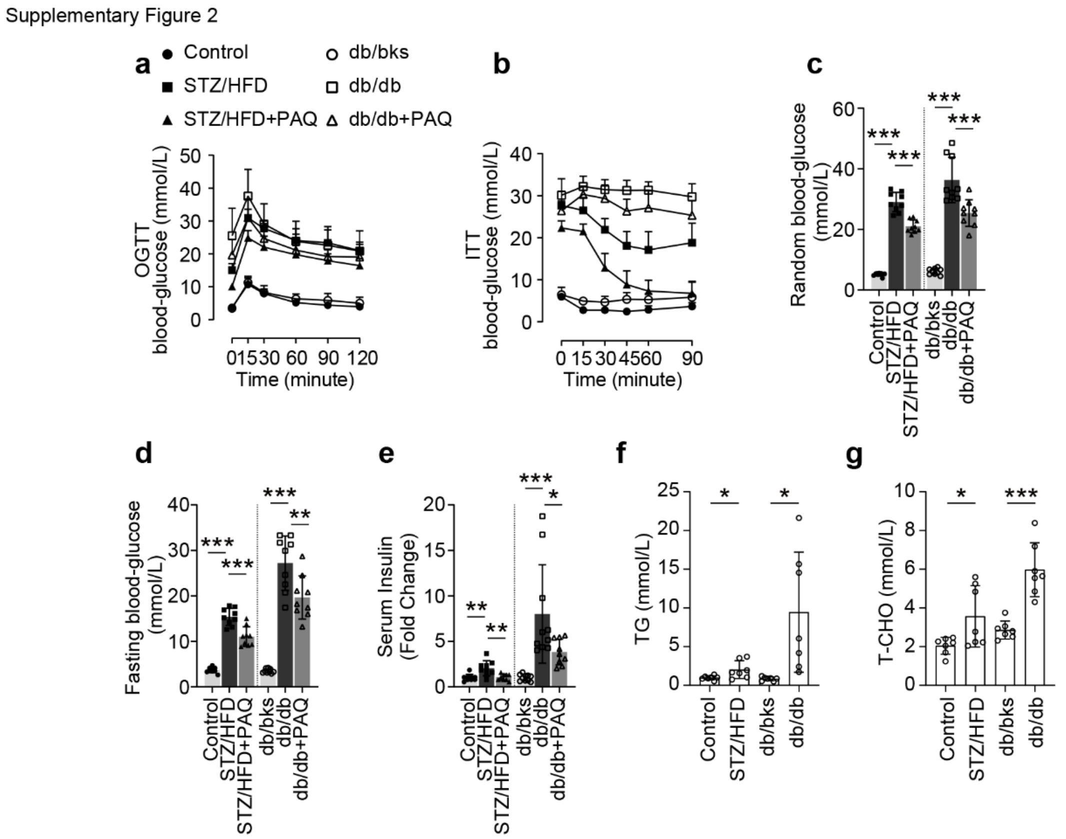

Supplementary Fig. 2 Blood glucose and lipid levels in diabetic mice.

a，OGTT (Oral Glucose Tolerance Test). **b,** ITT (Insulin Tolerance Test). **c,** random blood glucose. **d,** fasting blood glucose. **e,** insulin levels. **f,** total triglyceride (TG). **g,** total cholesterol (T-CHO). *$p < 0.05$, **$p < 0.01$, ***$p < 0.001$. All data are presented as mean ± SD. Statistical significance was determined by unpaired student's t-test (2f, 2g), or one-way ANOVA (2c-2e).

2

Supplementary Figure 3

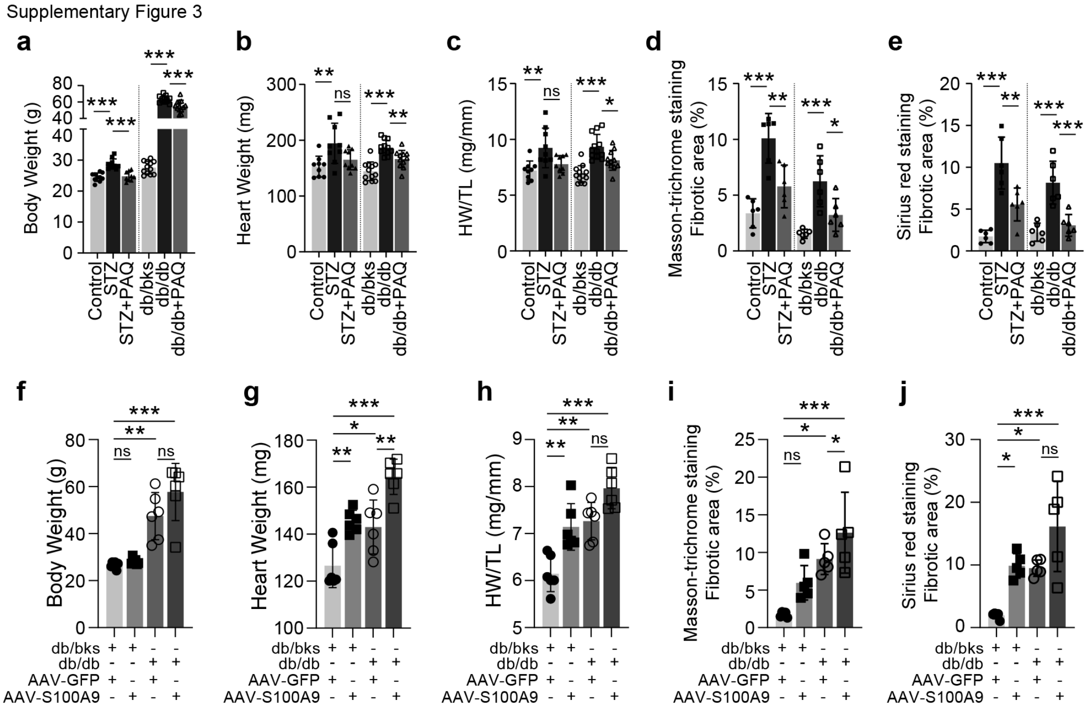

Supplementary Fig. 3 Quantification of heart morphology and fibrosis. Quantification of body weight, heart weight (HW), HW/TL(tibial length), Masson-trichrome staining, and Sirius red staining in STZ/HFD-induced or db/db diabetic mice (**a-e**) and mice with cardiac-specific overexpression of S100A9 (**f-j**). *$p < 0.05$, **$p < 0.01$, ***$p < 0.001$. All data are presented as mean ± SD. Statistical significance was determined by one-way ANOVA.

3

Supplementary Figure 4

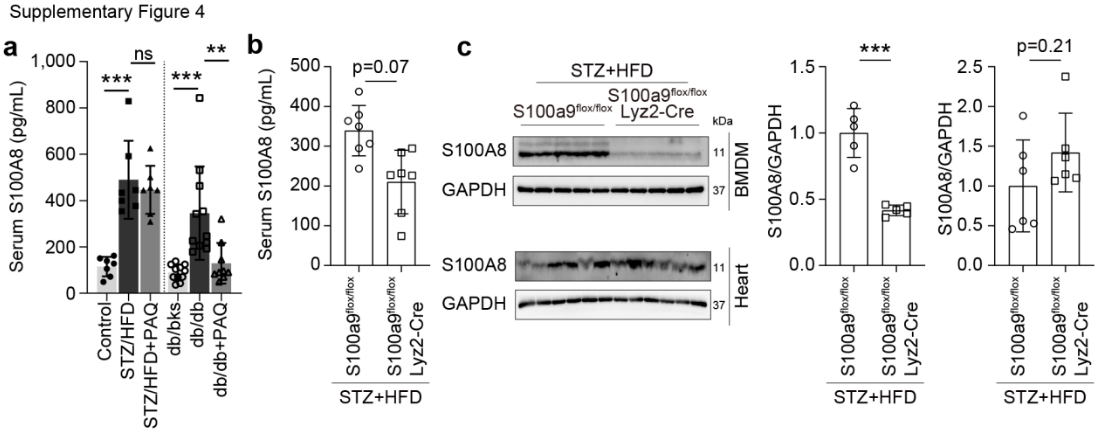

Supplementary Fig. 4 S100A8 expression in diabetic mice and macrophage specific S100a9 knockout mice.

a, Serum S100A8 concentration in diabetic mice (n=7, STZ group; n=9, db/db group). **b,** Serum S100A8 concentration in macrophage specific S100a9 knockout mice (n=7). c, S100A8 expression in BMDM (n=5) and heart (n=6) of macrophage specific S100a9 knockout mice. **p* < 0.05, ***p* < 0.01, ****p* < 0.001. All data are presented as mean ± SD. Statistical significance was determined by unpaired student's t-test (4b-4c), or one-way ANOVA (4a).

4

Supplementary Figure 5

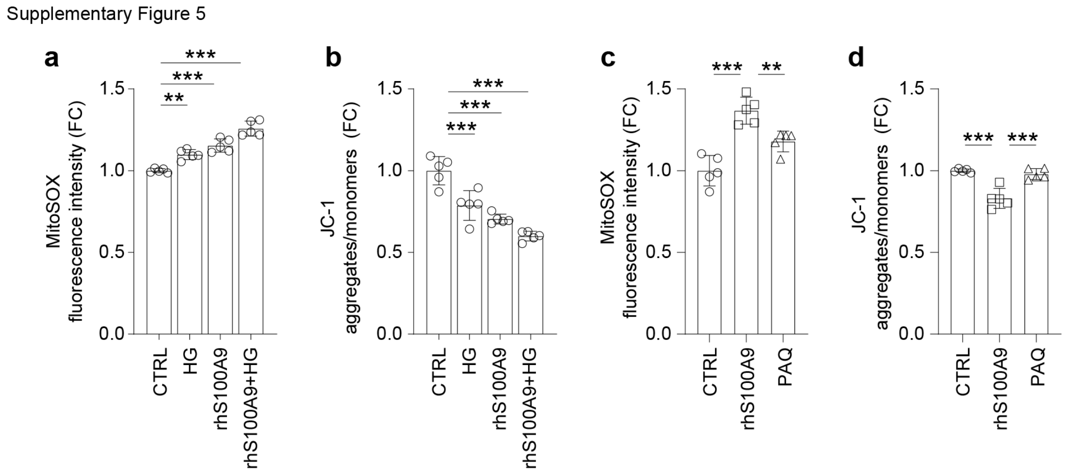

Supplementary Fig. 5 Quantification of MitoSOX and JC-1 staining in AC16 cells.

AC16 cells exposed to rhS100A9 (2μg/mL), high glucose (33mM), and/or paquinimod (PAQ, 20μM). **a** and **c,** MitoSOX staining. **b** and **d,** JC-1 staining. *$p < 0.05$, **$p < 0.01$, ***$p < 0.001$. All data are presented as mean ± SD. Statistical significance was determined by one-way ANOVA.

5
